# Antigenic Peptides Capable of Inducing Specific Antibodies for Detection of the Major Alterations Found in Type 2B Von Willebrand Disease

**DOI:** 10.1155/2013/590329

**Published:** 2013-07-18

**Authors:** Marina de Oliveira Paro, Cyntia Silva Ferreira, Fernanda Silva Vieira, Marcos Aurélio de Santana, William Castro-Borges, Maria Sueli Silva Namen-Lopes, Sophie Yvette Leclercq, Cibele Velloso-Rodrigues, Milton Hércules Guerra de Andrade

**Affiliations:** ^1^Departamento de Ciências Biológicas/DECBI, Instituto de Ciências Exatas e Biológicas/ICEB, Universidade Federal de Ouro Preto, Núcleo de Pesquisas em Ciências Biológicas/NUPEB, Campus Universitário Morro do Cruzeiro, 35400-000 Ouro Preto, MG, Brazil; ^2^Fundação Centro de Hematologia e Hemoterapia, Hemominas, Serviço de Pesquisa, Alameda Ezequiel Dias, 321, Bairro Santa Efigênia, 30130-110 Belo Horizonte, MG, Brazil; ^3^Fundação Ezequiel Dias, FUNED, Rua Conde Pereira Carneiro, 80, Bairro Gameleira, 30510-010 Belo Horizonte, MG, Brazil; ^4^Departamento Básico, Instituto de Ciências Biológicas, Universidade Federal de Juiz de Fora, Campus de Governador Valadares, Área da Saúde, Avenida Doutor Raimundo Monteiro de Resende, 330, Centro, 35010-177 Governador Valadares, MG, Brazil

## Abstract

Von Willebrand disease (VWD) is an inherited hemorrhagic disorder promoted by either quantitative or qualitative defects of the von Willebrand factor (VWF). The disease represents the most common human coagulopathy afflicting 1.3% of the population. Qualitative defects are subdivided into four subtypes and classified according to the molecular dysfunction of the VWF. The differential diagnosis of the VWD is a difficult task, relying on a panel of tests aimed to assess the plasma levels and function of the VWF. Here, we propose biochemical approaches for the identification of structural variants of the VWF. A bioinformatic analysis was conducted to design seven peptides among which three were representatives of specific amino acid sequences belonging to normal VWF and four encompassed sequences found in the most common VWD subtype 2B. These peptides were used to immunize mice, after which, peptide-specific immunoglobulins were purified. This resulted in four Ig preparations capable of detecting alterations in the subtype 2B VWD plus additional three antibody fractions targeting the normal VWF. The panel of antibodies could serve many applications among them (1) assessment of VWF: antigen interaction, (2) VWF multimer analysis, and (3) production of monoclonal antibodies against VWF for therapeutic purposes as in thrombotic thrombocytopenic purpura.

## 1. Introduction

Von Willebrand disease (VWD) is an inherited hemorrhagic disturbance related to quantitative and/or qualitative defects of the von Willebrand factor (VWF) [1, 2]. VWD prevalence varies between 0.8 and 2.0%, depending on the investigated population, being considered the most common coagulopathy afflicting humans [3]. 

The VWF is a multimeric plasma protein, composed of a varying number of 250 kDa monomers, which exhibits an essential role in primary haemostasis. Some of its reported functions are the attachment of platelets to subendothelial collagen at injured sites (platelet plug formation) and protection, binding, and transportation of coagulation factor VIII [4]. Mechanisms leading to the disease present themselves as highly diverse at the molecular level, giving rise to a variety of clinical outcomes. Through different laboratory criteria it is possible to identify three primary types of the disease [5]. Alterations on the plasma levels of VWF are associated with VWD types 1 and 3, whereas structural and functional defects of VWF result in VWD type 2 [3, 6–11]. VWD types 1 and 3 reflect, respectively, partial and complete deficiency of the VWF. VWD type 2 is also classified into four subtypes. In subtype 2A, there is enhanced platelet adhesion caused by the selective deficiency of high molecular mass multimers of VWF (HMW). In subtype 2B VWD, it is observed increased affinity of VWF by platelet glycoprotein Ib (GpIb) associated with loss of HMW-VWF and mild thrombocytopenia. In contrast, in subtype 2M, a pronounced reduction in platelet adhesion is described, even considering the relatively normal size of the VWF multimers. Variants of the VWF in the subtype 2N display reduced affinity by coagulation factor VIII (FVIII). Altogether these six VWD types correlate with diverse clinical outcomes each requiring adequate therapeutic interventions [4, 12]. Whilst some patients with type 2B VWD can be treated with desmopressin, patients who do not show a satisfactory response to this drug should receive VWF/FVIII-containing products [13, 14].

Considering the current difficulties associated with diagnosis of the qualitative defects of the VWF, the development of novel biochemical approaches that would allow detection of such molecular alterations is of great biotechnological interest. The possibility of a precise and direct diagnosis of qualitative defects in the VWF would certainly permit application of a better oriented medical approach to afflicted patients. In this context, it is worth emphasizing that antibodies capable of detecting structural variants of the VWF are not commercially available. The design of peptides for synthesis and further generation of antibodies to be employed in the identification of structural alterations in the VWF might therefore represent a convenient way forward. 

It has previously been shown that amino acid substitutions R1306W, R1308C, V1316M, and R1341Q account for 90% prevalence of the subtype 2B VWD [6, 8]. These mutations in the A1 domain of VWF are responsible for a “gain-of-function” defect allowing for an increased affinity of large multimers to platelets in the circulation [15]. In the present investigation we have generated a panel of antipeptide-specific antibodies useful at detecting the aforementioned structural variants of the VWF. 

## 2. Materials and Methods

### 2.1. Ethics Statement

All experiments involving mice were conducted according to approved guidelines for animal use and care defined by the Local Ethics Committee on Animal Experimentation (CEUA/UFOP). Healthy individuals were informed previously of the investigatory nature of this study, and after giving their consent, plasma samples were obtained under the procedures approved by the Local Ethics Committee from Hemominas Foundation, MG, Brazil. 

### 2.2. Selection of VWF Peptides for Synthesis

As stated previously the VWF amino acid substitutions R1306W, R1308C, V1316M, and R1341Q are collectively found in the majority of subtype 2B VWD patients [6]. In order to design signature peptides that would represent such mutations, *Homo sapiens *VWF sequences were retrieved from the International Society on Thrombosis and Haemostasis database, available at http://www.vwf.group.shef.ac.uk/. Predicted peptide sequences for synthesis obeyed the following criteria: (1) peptide size should be within the 8 to 10 mer range; (2) peptides should contain one aromatic residue for estimation of peptide yield after synthesis and HPLC purification (in the absence of an aromatic residue, this was added at the peptide C-terminal); (3) the location of the peptide sequences in the crystal structure of the VWF (available at http://www.pdbj.org/), revealed by the Swiss-Pdb Viewer 3.7, should demonstrate their exposure to the solvent, meaning that highly hydrophobic peptide sequences were not considered in this study. 

The selected peptide sequences were then aligned to *Mus musculus* VWF sequence using ClustalW2 (http://www.ebi.ac.uk/Tools/msa/clustalw2/) to reveal species specific amino acid differences as predictors for successful production of anti-VWF peptide antibodies in mice. 

### 2.3. Synthesis, Characterization, and Production of Polyclonal Antibodies against VWF Peptides

Peptides selected for synthesis were representatives of the normal and altered versions of the VWF found in the major subtypes of type 2 VWD (Table 1). Peptides were synthesized using the solid-phase protocol essentially as described by Merrifield [16]. Briefly, the support matrix consisted of Rink Amide Resin HL (Merck, Germany) at 0.78 mmol/g for an expected maximum yield of 40 *μ*M of peptides per synthesis, using Fmoc-derivatized amino acids. Synthetic peptides were then purified via reversed-phase chromatography using a C18 column (Shim-pack CLD-ODS Shimadzu, Japan) on a Shimadzu HPLC system. Selected peptide peaks were recovered for analysis through direct injection onto an electrospray-operating mass spectrometer (LCMS-IT-ToF, Shimadzu, Japan) in positive ionization mode and capillary voltage set to 4,500 V. Mass spectrometric data were acquired over 10 ms, after which *m/z* values obtained were compared with the expected molecular masses for the synthesized peptides. 

Synthetic peptides representatives of both normal and altered VWF were individually coupled to the highly immunogen carrier protein keyhole limpet hemocyanin (KLH, Sigma) at 1 : 1 ratio (1 mg peptide: 1 mg KLH) using glutaraldehyde as the crosslinking agent [17]. Polyclonal anti-KLH peptide antibodies were raised in male *Swiss* mice aged ten weeks. Immunization regimen consisted of three intraperitoneal administrations of 50 *μ*g of the KLH-peptide conjugate prepared in 10% aluminium hydroxide as adjuvant, each at a 15 days interval. Blood was withdrawn after 45 days post-immunization. 

### 2.4. Immobilization of Synthetic Peptides on Sepharose-4B and Purification of Anti-VWF Specific Immunoglobulins

Affinity columns (0.2 mL) containing immobilized synthetic peptides, at approximately 10 mg/mL, were produced as described previously [18]. The antisera from mice immunized with KLH coupled to altered VWF peptides were loaded individually in affinity columns bearing the corresponding normal versions of the VWF peptide. This procedure aimed the subtraction of antibodies targeting the normal VWF. Approximately 100 *μ*L of the unbound fraction was collected for analysis during the first chromatographic step, whilst the remaining were immediately submitted to further six rounds of chromatography to guarantee complete removal of antinormal VWF antibodies. For each chromatographic step bound fractions were eluted in 5 mL 0.1 M glycine pH 2.6 and collected in tubes containing 5 mL of 0.4 M Tris-HCl pH 8.0 for pH neutralization. Alkaline phosphatase labeled anti-mouse IgG, at 1 : 2000 dilution, was used to detect the presence of IgGs found in both nonretained and retained fractions from each chromatographic step, using the western blotting technique [19]. 

The next approach involved confirmation that antibodies targeting the normal VWF have been thoroughly subtracted after the six passages in affinity columns containing immobilized altered VWF peptides. For this purpose, 1 mL of pooled plasma from human healthy donors (*n* = 5) was first precipitated in 20% ethanol to enrich for normal VWF. Approximately 10 *μ*g aliquots of the precipitated plasma were resuspended in protein loading buffer and loaded in three different lanes for separation on a 7% SDS-PAGE. The gel was transferred to a PVDF (polyvinylidene fluoride) membrane and each lane western blotted with either a 100 *μ*L fraction obtained from the crude antisera or the two 100 *μ*L non-retained fractions recovered from the first and sixth chromatographic steps. 

The combined eluates from each chromatographic step containing immunoglobulins targeting a given altered VWF peptide were lyophilized, resuspended in 1 mL of phosphate buffer pH 7.4, and individually loaded onto the Sepharose-4B affinity column bearing the respective altered VWF peptide. Bound IgGs targeting the altered VWF peptide were eluted exactly as described above. 

### 2.5. Production of Carrier Albumins Bearing Altered VWF Synthetic Peptides and Their Recognition by Anti-VWF Specific Antibodies

Aiming to produce a model sample for detection of altered VWF through the western blotting technique, purified albumin was conjugated to two representative synthetic peptides (SQKWVRMA and RPSELQRY, Table 1) [20] at a 1 : 1 ratio, respectively. Briefly, to each 1 mg of the respective peptides it was added 17 *μ*L of N-N′diisopropylcarbodiimide, diluted 1 : 25 in dimethylformamide (DMF), and 20 *μ*L of 1 M N-hydroxysuccinimide (also diluted in DMF). Reaction proceeded during 5 min at room temperature, after which 50 *μ*L of 0.4 M sodium acetate (in DMF) was added. After further 2 min incubation, at room temperature, 1 mL of bovine serum albumin (BSA, Sigma), at 1 mg/mL, prepared in 0.05 M ammonium bicarbonate pH 7.5, was combined. Coupling reactions occurred during 1 h followed by dialysis of each preparation in 0.1 M ammonium acetate over 24 h. Peptide-derivatized albumins were lyophilized and resuspended in 100 *μ*L of saline. 

 Five *μ*g aliquots of peptide-conjugated albumin were loaded into three lanes and separated through 12% SDS-PAGE, followed by Coomassie staining. A replica gel was produced and transferred to a PVDF membrane. Antipeptide-specific IgG purified through affinity chromatography, at approximately 0.15 *μ*g/*μ*L, was used as the primary antibody, at a final dilution of 1 : 500. The reaction was allowed to proceed for 3 h at room temperature. Development of the reactive band was achieved using alkaline phosphatase labeled anti-mouse IgG (Sigma), at 1 : 2000 dilution for 2 h, followed by addition of the alkaline phosphatase substrates nitroblue tetrazolium/5-bromo-4-chloro-3-indolyl-phosphate (NBT/BCIP, Sigma) as per the manufacturer's instructions. 

## 3. Results and Discussion

In this study four amino acid sequences, here termed altered VWF peptides (MEWLRISY, MERLCISY, SQKWVRMA, and RPSELQRY), were synthesized for generation of antibodies in mice. These were intended for recognition of the most common qualitative defects of VWF found in subtype 2B VWD. Three additional peptide sequences MERLRISY, SQKWVRVA, and RPSELRRY, representatives of the respective normal versions of the VWF peptides, were also produced and immobilized onto Sepharose 4B, for depleting out immunoglobulins targeting the normal VWF from the antisera raised with the altered VWF peptides. Given that the corresponding *Mus musculus* VWF peptides are MERLHIS, SQKRIRVA, and RPSELRR, we anticipated that the observed amino acid differences between human and mice VWF would justify the peptides capabilities for generating specific antibodies in mice (Table 1). 

**Figure 1:**
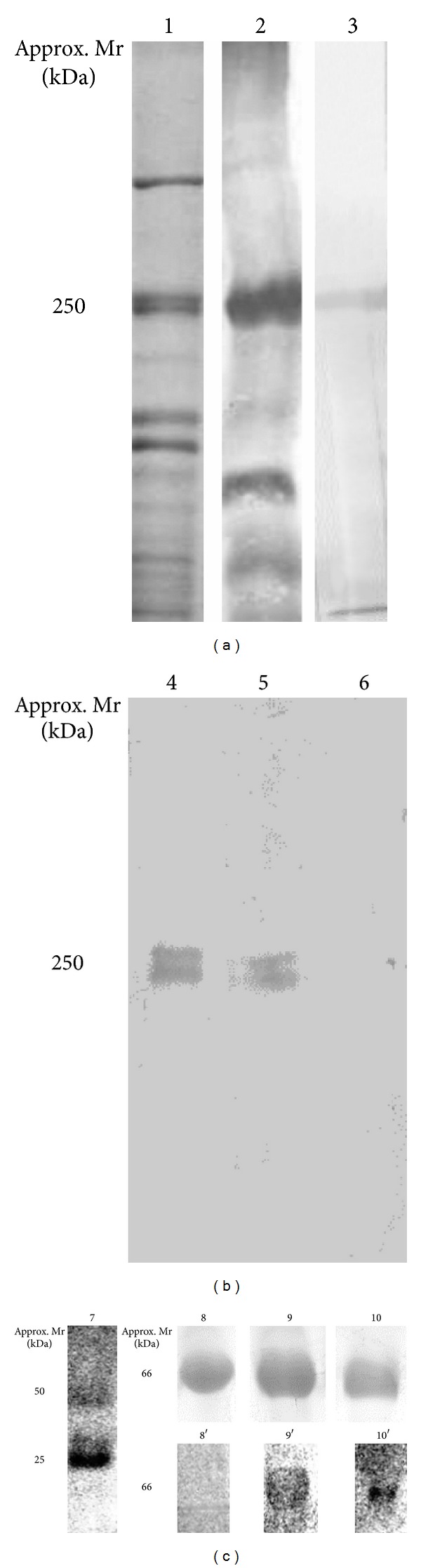
SDS-PAGE and western blotting approaches featuring the major steps involved in the production of a panel of anti-(KLH-peptide) antibodies for detection of VWD subtype 2B. (a) *Lane 1*, 10% SDS-PAGE profile of pooled human plasma after enrichment for normal VWF using ethanol precipitation; *Lane 2*, western blotting for detection of normal VWF, at approximately 250 kDa, using the commercially available antibody against VWF/FVIII—note the presence of additional protein bands being recognized, particularly at lower mass; *Lane 3*, a representative Western blotting reaction obtained with the generated panel of anti-(KLH-peptide) antibodies, targeting both normal and altered VWF. (b) Detection of normal VWf by western blotting following the subtractive affinity chromatography; *Lane 4*, detection of VWF prior to depletion of antibodies against the normal factor; *Lanes 5* and *6*, detection of VWF using the nonretained fractions from the first and sixth chromatographic steps, respectively—note that 6 column passages proved sufficient for complete removal of anti-(KLH-peptide) antibodies. (c) *Lane 7*, 10% SDS-PAGE profile representative of the eluates obtained after affinity purification of antibodies targeting specifically altered VWf peptides—note the presence of IgG heavy and light chains at approximately 50 and 25 kDa, respectively; *Lanes 8*, *9*, and *10*, 10% SDS-PAGE profile of nonderivatized BSA, BSA-(RPSELRR) and BSA-(SQKRIRVA), respectively; *Lanes 8*′, *9*′, and *10*′, corresponding western blotting reactions obtained using control sera, anti-(KLH-RPSELRR) and anti-(KLH-SQKRIRVA), respectively.

After immunization, the obtained antisera were tested for their ability to specifically recognize the VWF enriched from plasma samples of human healthy donors.  Figure 1(a), lane 3 is a representative reactivity obtained for all antisera raised with either the altered or normal versions of VWF peptides. A unique band at approximately 250 kDa was observed coinciding exactly with a major band, at the same mass, observed in Figure 1(a), lane 2. This was probed with a commercially available antiserum against VWF-FVIII. In contrast, the latter antibody preparation do recognize other protein bands (particularly of lower masses) in the sample (Figure 1(a), lane 2) revealing that the antisera generated herein are more suitable for specific recognition of the human VWF. 

The result obtained in Figure 1(a), lane 3 is a proof that the antisera produced using altered VWF peptides also contain IgGs targeting the normal VWF. Aiming to remove those immunoglobulins from the preparations, a subtraction experiment was conducted. Firstly, each individual antiserum obtained by immunization with a given altered VWF peptide was submitted to affinity chromatography on a Sepharose 4B column, containing the respective immobilized normal VWF peptide. Through collection of both non-retained and retained fractions during 6 chromatographic rounds, it was observed complete removal of IgGs targeting normal VWF. This can be visualized in Figure 1(b) (lanes 4, 5, and 6), which represents the enriched plasma probed with the antiserum prior to affinity chromatography (lane 4) or probed with the retained fractions from the first (lane 5) and sixth (lane 6) column passages. From this result we expected the immunoglobulins targeting specifically the altered VWF peptides to be found in the combined non-retained fractions. 

Isolation of anti-altered VWF specific immunoglobulins was achieved through new rounds of affinity chromatographies employing Sepharose 4B bearing immobilized altered VWF peptides. Bound immunoglobulins were eluted from each individual column and the presence of specific IgGs (heavy and light chains at approx. 50 and 25 kDa, resp.) confirmed by western blotting, using alkaline phosphatase labeled anti-mouse IgG (Figure 1(c), lane 7).

Given the experienced difficulty in obtaining a plasma sample from a VWD patient, for whom a qualitative alteration of the subtype 2B has been undoubtedly confirmed, we have engineered a model protein as a manner to test the diagnostic usefulness of our produced antisera. In this approach, nonconjugated BSA, BSA-SQKWVRMA plus BSA-RPSELQRY were separated using 1D 12% SDS-PAGE and the gel lanes western blotted with their respective peptide-specific antisera. As shown in Figure 1(c), nonconjugated albumin, which served as a control, was not detected by any of the anti-altered VWF peptide specific antiserum (Figure 1(c), represented by lane 8/8′). In contrast, the two derivatized albumins were recognized by the respective antisera generated against the two aforementioned altered VWF peptides (Figure 1(c), lanes 9/9′ and 10/10′). 

Finally, it is worth mentioning that the protocol used to produce the chimera albumins guarantees minimal coupling of the peptides. This should have resulted in a more realistic model samples being produced. Such information is of relevance considering that high sensitivity for detecting qualitative alterations in the VWF in human plasma is obviously desirable. 

## 4. Conclusions

In this study, a combination of bioinformatic analysis, peptide synthesis, and affinity chromatography allowed the generation of antipeptide specific antibodies capable of recognizing the most common qualitative defects associated with type 2B VWD. These should provide speed and innovation when potentially applied to the diagnosis of human VWD.

## Figures and Tables

**Table 1:** Proposed synthetic peptides for detection of the major qualitative alterations found in VWD type 2B.

Major qualitative alterations found in VWD type 2B	Designed 8 mer peptides for synthesis	Theoretical [M + H]^+^	Observed [M + H]^+^	Respective peptide from *M. musculus *VWF
R1306W	MEWLRISY	1097.54	1113.56*	MERLHIS
R1308C	MERLCISY	1014.47	1014.50	
	Respective peptide found in *H. sapiens *VWF: MERLRISY	1067.56	1082.57*	

V1316M	SQKWVRMA	1005.52	1020.54*	SQKRIRVA
	Respective peptide found in *H. sapiens *VWF: SQKWVRVA	973.55	972.57 [−1H^+^]	

R1341Q	RPSELQRY	1048.55	1048.57	RPSELRR
	Respective peptide found in *H. sapiens *VWF: RPSELRRY	1076.59	1076.62	

*Peptide mass containing an oxidized methionine.

## Abbreviations

VWF: Von Willebrand factor
VWD: Von Willebrand disease.
